# Lupus Enteritis and Rare Severe Manifestations of Lupus: Why Classification Criteria should not be used for Diagnosis

**DOI:** 10.31138/mjr.230625.lhe

**Published:** 2025-06-30

**Authors:** Antonis Fanouriakis

**Affiliations:** Rheumatology and Clinical Immunology, Attikon University Hospital, Medical School National and Kapodistrian University of Athens, Greece

**Keywords:** lupus enteritis, lupus, diagnosis, classification criteria

To underline the phenotypic heterogeneity of systemic lupus erythematosus (SLE), it is occasionally said that patients with this disease are “like snowflakes”, each one different from the other. There are patients with a typical clinical picture, for example fever, a photosensitive rash, polyarthritis, and compatible serology, in whom diagnosis is usually straightforward. On the other hand, the diagnosis becomes much more challenging in patients who present with a dominant, often organ- or even life-threatening manifestation from a single organ system and very subtle -if any- findings from other organs. In such cases, especially in a patient without a prior diagnosis of SLE, and while immunologic results are pending, a physician should be aware that SLE should be included in the differential diagnosis, following the exclusion of competing, more common conditions. In this issue of the Mediterranean Journal of Rheumatology, two studies from India, a case report by Zope et al.^1^ and an observational study by Kotha et al.^2^ report on such a rare, but severe, manifestation of SLE, namely lupus enteritis. Remarkably, the importance of prompt recognition is highlighted by the compelling 31% mortality reported by Kotha et al.^2^ Gastrointestinal (GI) symptoms in general are not rare in SLE, but they are most often attributed to medication side-effects or superimposed infectious complications; the most common disease-related manifestation is oral ulcers. Less frequently, however, the GI tract can be involved by manifestations that present with an acute abdomen-like syndrome. These complications have been categorised by the British Isles Lupus Assessment Group (BILAG)^3^ and are, in order of increasing severity: i) abdominal serositis (presence of ascitic fluid), ii) lupus peritonitis (abdominal serositis presenting as acute abdomen with rebound tenderness/guarding), and iii) lupus mesenteric vasculitis (also known as lupus enteritis); the latter is the most serious GI manifestation in SLE, which can lead to bowel necrosis and/or perforation. The diagnosis of lupus enteritis is based on the characteristic abdominal computed tomography findings of target sign (bowel wall thickening with abnormal wall enhancement) and comb sign (engorgement of mesenteric vessels), which usually -but not always- are present in the context of generalised disease activity.^4^ Nevertheless, neither of these imaging findings is absolutely specific for lupus enteritis; even more, in the series of Kotha et al.,^2^ a target sign was present in less than 20% of patients!

In the report by Zope et al.,^1^ the patient had a known history of SLE; in the study by Kotha et al.,^2^ the mean interval between onset of SLE and the occurrence of enteritis was almost 2.5 years, but it is not specified what percentage of patients -if any- developed enteritis as a presenting manifestation of the disease, when the diagnostic challenge is maximum. It is important to note that, owing to their rarity, these serious GI manifestations of SLE are not included in any set of classification criteria for the disease.^5–7^ This is not unexpected, because classification criteria by definition need to balance specificity with sensitivity, thus do not typically include very rare manifestations of a disease (explaining also, for example, why only certain neuropsychiatric manifestations of SLE are included, from the wide array observed in lupus patients). Even though classification criteria are not developed for the purpose of diagnosis, it is well recognised that, in routine clinical practice, they are frequently used for diagnostic purposes, especially in patients with a complex or multi-systemic clinical picture, and by physicians with limited experience in SLE. In such circumstances, a patient without known SLE who presents with a single, severe manifestation, which is rare (in this case, lupus enteritis), cannot be classified with SLE, even in the presence of a typical lupus serology. In autoimmune rheumatic diseases, diagnosis needs an experienced physician and may also entail the notion of possibility (i.e., possible SLE), which can justify the decision to administer immunosuppressive treatment, following the exclusion of alternative diagnoses. This approach was suggested in a viewpoint article published more than 10 years ago, in an effort to overcome these inherent limitations of classification criteria.^8^

Outside diagnosis, how should lupus enteritis be treated? An additional problem with rare disease manifestations is the dearth of evidence to guide their management, since published experience originates mostly from anecdotal reports or case series. Accordingly, the infrequency of similar severe manifestations has left them out of the management recommendations published by the European Alliance of Associations for Rheumatology, owing to this lack of evidence.^9^ More recently, the published summary of the American College of Rheumatology guidelines give a conditional recommendation for any immunosuppressive therapy (conventional or biologic) in combination with glucocorticoids, for any “systemic vasculitis” attributed to SLE, including mesenteric vasculitis. If the condition is considered life-threatening, addition of plasma exchange or IVIG is conditionally recommended, again with very low quality of evidence. Very recently, to aid physicians who encounter these rare severe manifestations, a joint expert consensus manuscript by the ERN ReCONNET, SLICC, and SLEuro was released, with proposed treatment regimens for a variety of rare manifestations.^8^ In the case of lupus enteritis, and based on severity stratification, most experts favoured the use of IV cyclophosphamide (for severe) or mycophenolate mofetil (for less severe cases).

In conclusion, the two reports in the current issue of the Mediterranean Journal of Rheumatology remind us of the protean manifestations of SLE and, accordingly, the inherent limitations of any set of classification criteria, when used for diagnosis. Physicians caring after patients with lupus and, even more, hospitalists working in Emergency Rooms and Internal Medicine wards should be aware of these rare, yet serious, manifestations of this heterogeneous disease. In other words, to diagnose a condition, one has first to be aware of it.

## CONFLICT OF INTEREST

The author declares no conflict of interest.
